# FAMILIAL DYSPHAGIA AND ITS ASSOCIATION WITH EOSINOPHILIC ESOPHAGITIS

**DOI:** 10.51894/001c.123113

**Published:** 2024-08-30

**Authors:** Ananya Varre

**Affiliations:** 1 College of Osteopathic Medicine Michigan State University

## 91

### INTRODUCTION

Eosinophilic esophagitis (EoE) is diagnosed by the presence of over 15 eosinophils per high power field (hpf), resulting from genetic and environmental factors with rising prevelance in the past two decades. Symptoms for EoE include dysphagia, vomiting, and food impaction. Untreated EoE correlates with esophageal stricture development, highlighting the importance of timely intervention. Schatzki rings (SR), submucosal circumferential rings in the distal esophagus, are associated with EoE. Recent studies suggest a familial connection, with older family members presenting with dysphagia or SR while younger generations are diagnosed with EoE. This familial link and progression are observed in three patients from the same family, reinforcing the proposed connection between EoE, SR, and familial patterns.

### CASE DESCRIPTION

Exploring three family members, Patient A (24-year-old male), Patient B (68-year-old great aunt), and Patient C (deceased great grandmother), who share a history of dysphagia and stricture. Patient A: A 24-year-old male with dysphagia to solids who required emergency interventions for food impaction. His esophageal biopsy showed > 50/hpf, prompting a diagnosis of EoE. Patient B: A 68-year-old female with severe dysphagia to solids since her early 50s. Multiple dilations and EGDs were performed, revealing a nonobstructing SR at the gastroesophageal junction. Biopsies for EoE were negative (< 15/hpf). She also had a hiatal hernia and periampullary diverticulum, treated with pantoprazole. Patient C: Deceased female patient with esophageal stricture, undergoing multiple dilation procedures during adulthood.

### DISCUSSION/CONCLUSIONS

Our results suggest a correlation between SR and EoE within a family. Patient A presents with EoE and SR, indicating a potential progression to SR from EoE. As emphasized in previous studies, the chronic nature of EoE can lead to irreversible structural changes in the esophagus such as ongoing dysphagia or SR like in patients B and C. Monitoring patient A’s progression will be crucial to determine if EoE transforms into stricture and SR without EoE as they age. Our study provides insights on the genetic influence of stricture, SR, and EoE across three generations. The familial link between dysphagia or SR in older family members progressing from EoE in younger family members reinforces the genetic component.

